# Critical illness and flat batteries

**DOI:** 10.1186/s13054-017-1913-9

**Published:** 2017-12-28

**Authors:** Mervyn Singer

**Affiliations:** Bloomsbury Institute of Intensive Care Medicine, Cruciform Building, University College London, London, WC1E 6BT UK

## Abstract

An exaggerated, dysregulated host response to insults such as infection (i.e. sepsis), trauma and ischaemia-reperfusion injury can result in multiple organ dysfunction and death. While the focus of research in this area has largely centred on inflammation and immunity, a crucial missing link is the precise identification of mechanisms at the organ level that cause this physiological-biochemical failure. Any hypothesis must reconcile this functional organ failure with minimal signs of cell death, availability of oxygen, and (often) minimal early local inflammatory cell infiltrate. These failed organs also retain the capacity to usually recover, even those that are poorly regenerative. A metabolic-bioenergetic shutdown, akin to hibernation or aestivation, is the most plausible explanation currently advanced. This shutdown appears driven by a perfect storm of compromised mitochondrial oxidative phosphorylation related to inhibition by excessive inflammatory mediators, direct oxidant stress, a tissue oxygen deficit in the unresuscitated phase, altered hormonal drive, and downregulation of genes encoding mitochondrial proteins. In addition, the efficiency of oxidative phosphorylation may be affected by a substrate shift towards fat metabolism and increased uncoupling. A lack of sufficient ATP provision to fuel normal metabolic processes will drive downregulation of metabolism, and thus cellular functionality. In turn, a decrease in metabolism will provide negative feedback to the mitochondrion, inducing a bioenergetic shutdown. Arguably, these processes may offer protection against a prolonged inflammatory hit by sparing the cell from initiation of death pathways, thereby explaining the lack of significant morphological change. A narrow line may exist between adaptation and maladaptation. This places a considerable challenge on any therapeutic modulation to provide benefit rather than harm.

## Background

A wide range of insults, including infection, trauma, pancreatitis and ischaemia-reperfusion injury, can trigger a dysregulated host response that can lead, via a (likely) common pathway, to multi-organ failure and death. The top end of the pathway is reasonably well characterized [1, 2]. Innate immune receptors known as pattern recognition receptors (PRRs; e.g. Toll-like receptors (TLRs) and nucleotide-binding oligomerization domain (NOD)-like receptors (NLRs)) are activated either by microbial PAMPs (‘pathogen-associated molecular patterns’) or host cellular components known collectively as DAMPs (‘damage-associated molecular patterns’). Examples of PAMPs include endotoxin, lipoteichoic acid and bacterial or viral DNA or RNA, while DAMPs (released during cell damage or death) include DNA, mitochondria, uric acid and heat shock proteins. Activation of PRRs increases transcription of a wide range of both pro- and anti-inflammatory cytokines and production of multiple other mediators such as the eicosanoids and reactive oxygen species, including nitric oxide. Apart from activating the immune response, hormonal, metabolic, bioenergetic and other pathways are also modulated in either positive or negative directions [1, 2].

The innate immune response has been the primary focus of research, particularly in relation to infection. However, much less attention has been paid to identification of mechanisms that result in organ dysfunction/failure, especially affecting those organs removed from the site of the insult. A series of clinical observations in both patients and animal models add further intrigue and complexity. The histology of these failed organs show minimal, if any, cell death, even when examined soon after the patient’s demise [3]. In survivors, the failed organs usually recover sufficient functionality within days to weeks such that a long-term requirement for organ support is rarely needed [4]. This occurs even in organs with poor regenerative capacity. Furthermore, after adequate resuscitation, levels of tissue oxygen tension in various organ beds are normal or even elevated [5–8], indicating an availability of oxygen that meets or even exceeds cellular metabolic demands. These findings are all incompatible with the concept of tissue hypoxia resulting in ischaemic injury and cell damage as the predominant pathophysiological mechanism.

A paradigm is thus needed that can embrace this seemingly paradoxical combination of organ dysfunction occurring in the absence of significant structural damage yet provision of adequate oxygen. Cellular metabolic shutdown is a concept that satisfies these above observations. This shutdown is analogous to hibernation or aestivation where the normal functioning of the organism is lost as part of a process that preserves cell integrity though at the expense of functionality. Oxygen consumption falls in such situations in conjunction with a fall in metabolic rate. Patients with sepsis and trauma are ‘hypermetabolic’ in the early stages of the insult as the body initially fights to defend itself. However, with a prolonged insult there is a progressive reduction in oxygen consumption which, in severely affected patients, can fall to near-baseline levels for a healthy person [9, 10]. A rebound increase in metabolism occurs in the recovery phase, with metabolic rate rising > 50% above normal [9, 11].

Several mechanisms can potentially induce this metabolic shutdown. These may relate to a direct effect on metabolism with repurposing of metabolic pathways, and/or to secondary effects related to a progressive decrease in energy substrate (ATP) availability and a consequent metabolic shutdown. If metabolic processes continue without sufficient ATP to fuel them, cellular ATP levels will fall and, beyond a certain threshold, can trigger activation of cell death pathways. To avoid suicide the cell can attempt to compensate by switching off metabolic processes unconnected with survival that can maintain ATP levels above the critical threshold [12]. There is significant control over the rates of individual ATP consumers by energy supply [13]. The hierarchy consists of protein and RNA/DNA synthesis being the most sensitive to energy supply, followed by sodium and then calcium cycling across the plasma membrane, and mitochondrial proton leak being the least sensitive. In consequence, processes relating to the usual functionality of the cell, such as protein synthesis, can be downregulated or even abandoned. Conversely, pathways needed to maintain cellular integrity are retained, such as Na^+^K^+^ATPase activity that preserves membrane potential, transports substrates and electrolytes into and out of the cell and prevents cell swelling and lysis.

**Fig. 1:**
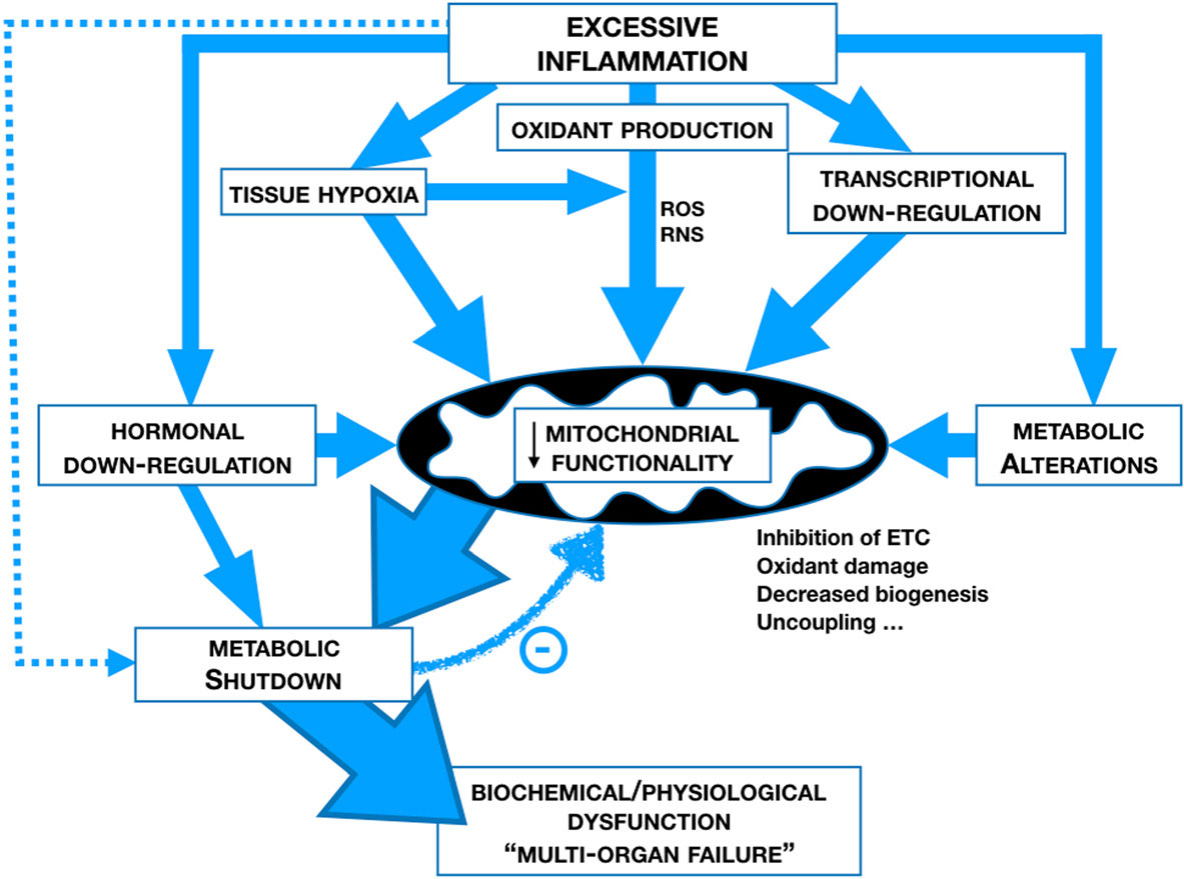
Mechanisms of mitochondrial and metabolic shutdown. *ETC* electron transport chain, *RNS* reactive nitrogen species, *ROS* reactive oxygen species

As mitochondrial respiration is primarily responsible in most cell types for provision of ATP, this organelle is likely integral to the process of metabolic shutdown through a reduction in energy availability. Several factors associated with a prolonged and/or severe stressful insult can trigger this shutdown, and these may be synergistic (Fig. 1). Such factors include:

1. *Prolonged inflammation with excessive production of mediators such as nitric oxide and other reactive species.* Mitochondria are the predominant source of reactive oxygen species (ROS) production in the body and, in health, play an important role in signalling. In excess, however, ROS have a direct inhibitory effect on mitochondrial respiration, either through direct inhibition of the electron transport chain or damage to the organelle when mitochondrial antioxidant defences are overwhelmed [14–17]. Routinely used treatments in the critically ill, such as bacteriocidal antibiotics [18], catecholamines [19] and sedatives [20], may also inhibit mitochondrial respiration.
2. *Tissue hypoxia, especially before adequate resuscitation.* This hypoxia is of insufficient magnitude to trigger cell death pathways yet severe enough to compromise normal functioning of the cells. Consequential to the ongoing oxygen debt, the cell responds by an adaptive compensatory reduction in metabolism to balance supply and demand. There are further corollaries of tissue hypoxia. As oxygen and nitric oxide compete for the same binding site on complex IV (cytochrome oxidase), the last component of the electron transport chain, a decrease in local oxygen concentration will enhance the inhibitory effect of nitric oxide described above [21].
3. *Mitochondrial respiration, which is modulated by various hormones and transcription factors.* Thyroid hormone, for example, has profound effects on ATP synthesis and turnover [22, 23]. It can also activate uncoupling of oxidative phosphorylation (see below); this mechanism may be responsible for some of its hypermetabolic effects. Complex relationships are reported between mechanisms of mitochondrial proton leak, production of reactive oxygen species and thyroid status. However, with prolonged and severe illness, there is decreased availability of active thyroid hormone (low T3 syndrome, sick euthyroid syndrome, non-thyroidal illness syndrome), the degree of which is prognostic [24]. This may impact on mitochondrial function and ATP turnover during critical illness.
4. *Decreased turnover of healthy, functional mitochondria (biogenesis).* Mitochondrial biogenesis must keep pace with mitophagy (processes that eliminate dysfunctional mitochondria) to maintain mitochondrial density. Several mechanisms may all conspire to decrease mitochondrial biogenesis in sepsis. In a pioneering study, Calvano and colleagues administered endotoxin to healthy volunteers and noted a generalized downregulation of gene transcripts encoding mitochondrial proteins, including those within the electron transport chain [25]. Transcription factors such as PGC-1alpha, the ‘master regulator’ of biogenesis, is reduced in animal models of sepsis and in eventual human non-survivors [26, 27]. Of note, the reduction in mitochondrial turnover may be iatrogenically compounded by bacteriostatic antibiotic therapy that impacts negatively upon biogenesis through decreasing protein synthesis [28].
5. *Uncoupled respiration with production of heat rather than ATP.* Most of the oxygen used by the body is consumed by mitochondria, predominantly for generation of ATP—so-called ‘coupled’ respiration. A proportion is uncoupled, whereby the proton gradient created by electron transfer down the electron transport chain is dissipated, and the energy is ‘lost’ as heat [29]. The precise amount of oxygen utilised by uncoupled respiration is uncertain. Ex vivo studies in different rat tissues suggests this proton leak varies from 15% in heart to as high as 50% in skeletal muscle [29]. Whether this increases in sepsis and other critical illness is not yet established [30], although a recent study showed an increase in uncoupling protein-1 in white adipose tissue after human burn injury [31]. This may explain, at least in part, pyrexia, especially as other mechanisms of heat production, such as muscular activity and food breakdown, are reduced in a sick patient. However, two corollaries of increased uncoupling are a reduction in ATP for fuelling metabolism yet also a reduction in mitochondrial membrane potential that may decrease production of damaging ROS and thus offer protection [32, 33].

Circulating humoral factors likely play a role. Belikova et al. [34] studied the impact of peripheral blood mononuclear cells (PBMCs) incubated in healthy volunteer plasma or plasma pooled form septic patients. While overall oxygen consumption decreased by approximately a third, the proportion of respiration coupled to ATP production fell from 89 to 55%. Likewise, Boulos et al. found septic plasma had a depressant effect on endothelial cell oxygen consumption and ATP levels when incubated ex vivo, and this could be prevented by nitric oxide synthase inhibition [35].

The literature is, however, conflicted when analysing the presence of mitochondrial dysfunction in critical illness, especially with respect to animal models [36]. This disparity does not consider the impact of time or illness severity, nor inter-organ or inter-species differences. Short-term models often fail to show an effect, or even demonstrate increased activity, reflecting the need to use better representative models of the human condition [37].

It is feasible that the above changes represent an adaptive response to prolonged stress. The kidney is a useful exemplar organ to argue this case. Acute kidney injury and failure are commonplace in critical illness yet acute tubular necrosis is an unusual finding in both septic patients and animal models [38]. Indeed, awareness of this marked histological normality has been reported for critical illness in general for 60 years [39]. Forty years ago, Thurau and Boylan [40] argued that acute renal failure represented acute renal success; most of the workload of the kidney relates to reabsorption of approximately 98% of glomerular filtrate; sparing an ischaemic and/or stressed kidney with this heavily energy-dependent task of reabsorbing large volumes of salt and water is thus a logical means of offering protection. Short-term shutdown translates into an ability to recover function in those who survive their critical illness.

There are many biological equivalents (torpor, dormancy, aestivation, hibernation) where metabolism switches off within hours in the face of an extreme and prolonged stress such as cold, heat, food shortage or drought. An approximate 40–70% of the decrease in metabolic rate is considered due to active metabolic suppression, with the remainder related to passive thermal effects as core temperature falls [41]. The dormouse can drop its core temperature to ambient and its metabolic rate by 90% within 3 h [42]. Mitochondrial respiration is suppressed and this occurs quickly during entrance into torpor when body temperature is still high. The rapidity of this response may reflect epigenetic modifications of mitochondrial electron transport chain complex activity, e.g. by phosphorylation or acetylation.

Another potential mechanism of metabolic suppression in sepsis may relate to inhibition of mitochondrial activity through increased production of the endogenous gases nitric oxide, carbon monoxide and hydrogen sulphide. This production can happen rapidly, within minutes to hours. We reported rapid falls in core temperature in septic mice given a faecal peritonitis insult, especially in eventual non-survivors [43]. Within 6 h the core temperature had fallen by 8 °C. Oxygen consumption fell by 38% within 2 h, and by 80% at 22 h. Conversely, in septic rats, temperature and oxygen consumption were initially maintained, although a pre-terminal fall in oxygen consumption was routinely seen commencing 6–8 h before death. It is conceivable that the maintained oxygen consumption in rats as well as humans is reprioritised towards heat production in sepsis, generating pyrexia but at the expense of fuelling normal processes. Of note, histological and biochemical changes analogous to those seen in hibernation have been described in septic mouse myocardium [44]. Myocardial hibernation is well recognized in humans in ischaemic hearts where persisting hypoperfusion results in decreased myocardial contractility to match substrate supply, but which recovers on reperfusion. The parallels between hibernation-like states in animals with critical illness in humans are striking and potentially translatable [45]. The underlying mechanisms are not necessarily duplicated as evolutionary pressures may have determined upregulation of different pathways. However, as Boutillier commented, “the key to (cell) survival (in hypoxia and hypothermia) lies in an inherent ability to downregulate cellular metabolic rate to new hypometabolic steady states in a way that balances the ATP demand and ATP supply pathways” [46]. A major challenge in patient management is to recognize when our efforts to intervene, which are often predicated on trying to achieve ‘normality’ of physiological and biochemical values, are counter-productive to the body’s attempts to adapt and, ultimately, injurious.

Recovery from organ dysfunction is preceded by evidence of increased mitochondrial biogenesis in both long-term animal models [26] and patients [27]. This may simply be epiphenomenal; interventional studies demonstrating improved survival rates or faster resolution of organ failure through stimulation of biogenesis are still lacking. Nevertheless, recovery in human sepsis and trauma is associated with marked increases in metabolic rate as the body switches back to anabolism and repair processes [9, 11]. A failure to thrive—leading to a persistent inflammation, immunosuppression and catabolism syndrome (PICS) [47]—may potentially be caused by an ongoing failure of mitochondrial/metabolic recovery.

In conclusion, while pathways leading to inflammation and immune activation/suppression have been extensively studied in sepsis, the precise mechanisms underlying multi-organ failure remain unknown. Circumstantial evidence strongly points to a metabolic shutdown triggered by a failure of mitochondrial ATP generation and/or cellular reprioritisation of energy utilisation. Targeted modulation of these processes has yet to show improved outcomes but the concept is appealing [12, 45]. Nevertheless, timing is likely to be critical as the body may object to metabolism being driven up prematurely [48].
